# Race, ethnicity, poverty and the social determinants of the coronavirus divide: U.S. county-level disparities and risk factors

**DOI:** 10.1186/s12889-021-11205-w

**Published:** 2021-06-29

**Authors:** Laura J. Samuel, Darrell J. Gaskin, Antonio, J. Trujillo, Sarah L. Szanton, Andrew Samuel, Eric Slade

**Affiliations:** 1 Johns Hopkins University School of Nursing, 525 North Wolfe St., Rm 426, Baltimore, MD 21205 USA; 2 Department of Health Policy and Management, Johns Hopkins Bloomberg School of Public Health, Baltimore, USA; 3 Department of International Health, Johns Hopkins Bloomberg School of Public Health, Baltimore, USA; 4 Department of Economics, Loyola University Maryland Sellinger School of Business, Baltimore, USA

**Keywords:** Coronavirus, Social determinants of health, Health disparities

## Abstract

**Background:**

Communities with more Black or Hispanic residents have higher coronavirus rates than communities with more White residents, but relevant community characteristics are underexplored. The purpose of this study was to investigate poverty-, race- and ethnic-based disparities and associated economic, housing, transit, population health and health care characteristics.

**Methods:**

Six-month cumulative coronavirus incidence and mortality were examined using adjusted negative binomial models among all U.S. counties (*n* = 3142). County-level independent variables included percentages in poverty and within racial/ethnic groups (Black, Hispanic, Native American, Asian), and rates of unemployment, lacking a high school diploma, housing cost burden, single parent households, limited English proficiency, diabetes, obesity, smoking, uninsured, preventable hospitalizations, primary care physicians, hospitals, ICU beds and households that were crowded, in multi-unit buildings or without a vehicle.

**Results:**

Counties with higher percentages of Black (IRR = 1.03, 95% CI: 1.02–1.03) or Hispanic (IRR = 1.02, 95% CI: 1.01–1.03) residents had more coronavirus cases. Counties with higher percentages of Black (IRR = 1.02, 95% CI: 1.02–1.03) or Native American (IRR = 1.02, 95% CI: 1.01–1.04) residents had more deaths. Higher rates of lacking a high school diploma was associated with higher counts of cases (IRR = 1.03, 95% CI: 1.01–1.05) and deaths (IRR = 1.04, 95% CI: 1.01–1.07). Higher percentages of multi-unit households were associated with higher (IRR = 1.02, 95% CI: 1.01–1.04) and unemployment with lower (IRR = 0.96, 95% CI: 0.94–0.98) incidence. Higher percentages of individuals with limited English proficiency (IRR = 1.09, 95% CI: 1.04–1.14) and households without a vehicle (IRR = 1.04, 95% CI: 1.01–1.07) were associated with more deaths.

**Conclusions:**

These results document differential pandemic impact in counties with more residents who are Black, Hispanic or Native American, highlighting the roles of residential racial segregation and other forms of discrimination. Factors including economic opportunities, occupational risk, public transit and housing conditions should be addressed in pandemic-related public health strategies to mitigate disparities across counties for the current pandemic and future population health events.

**Supplementary Information:**

The online version contains supplementary material available at 10.1186/s12889-021-11205-w.

## Background

Despite widespread attention to United States (U.S.) racial and poverty-based coronavirus disparities and early evidence of disparities across communities, national evidence is limited in examining potential underlying explanatory factors. Counties with higher proportions of Black or Hispanic residents have higher rates of coronavirus incidence and mortality than counties with relatively more White residents [1–4]; a disproportionate number of Black, Hispanic and Native American individuals in the US have been hospitalized for coronavirus [5, 6] and died of it [7, 8]. Although counties with higher rates of uninsured individuals and household crowding have been shown to have higher incidence [2, 4], there are gaps in examining other social determinants of health and population health and health care characteristics. Also, prior studies have not accounted for other relevant demographic factors, including population age, sex, or non-White and non-Black racial groups. This information is needed to identify community characteristics that are potential risk factors of disparities relevant to the novel coronavirus.

Disparities are likely attributable to the persistent effects of residential racial segregation and concentrated poverty, which likely influence multiple relevant risk factors [9, 10], including economic, housing, population health and health care characteristics. As examples, residing in crowded or multi-generational housing because of economic hardship or cultural factors [11] or the use of public transit may facilitate the spread of the disease in the community [12]. Also, economic pressures arising from single parenting, limited English proficiency or residing in communities with higher unemployment or housing costs may cause individuals to continue working even if they risk exposure to coronavirus or are ill. Likewise, individuals with relatively lower education working in low-skills jobs may not have remote work options. In addition, individuals living on incomes below poverty, or racial and ethnic minorities may have a higher risk of severe illness or mortality [13] because of their greater burden of underlying chronic diseases [14–17], and lack of health care access [18].

The need to identify and describe communities that are disproportionately affected by coronavirus is pressing. Such information could be used by state and federal agencies to test, distribute resources, provide guidance to communities and develop population health strategies. Therefore, this study first aims to characterize county-level racial, ethnic and poverty-based disparities in the introduction and burden of coronavirus cumulative incidence and mortality. Second, this study seeks to test the hypothesis that economic characteristics, housing and transit characteristics, and population health and health care access characteristics are associated with coronavirus incidence and mortality. We hypothesize that accounting for these characteristics attenuates the extent of the disparities, suggesting that they may partially account for disparities (see Supplemental Fig. 1). The second hypothesis draws upon tested theories about mechanisms that underlie both racial- and poverty-based disparities of other health outcomes [9, 19, 20] to identify potential risk factors for the novel coronavirus. Specifically, Fundamental Cause Theory [9] and Ecosocial Theory [19] highlight the importance of economic factors that influence exposure to environmental factors, including the housing and transit environments in which people live their daily lives, and influence access to health care and health promoting resources that shape population health profiles in communities.

## Methods

### Sample and outcome data

All 3142 U.S. counties within the 50 states and Washington DC were included in the study. The study interval included the first 6 months (180 days: January 22, 2020 –July 19, 2020) of the U.S. epidemic. Six-month cumulative coronavirus case incidence, mortality and days since identification of a county index case data were obtained from the Hopkins’ Center for Systems Science and Engineering [21]. Incidence included presumptive positive and probable cases and deaths include confirmed and probable deaths. Coronavirus cases that occurred in U.S. protectorate areas (*n* = 12,697) or that could not be assigned to a county Federal Information Processing Standard code (*n* = 40,969) were excluded, leaving 3,719,594 cases of coronavirus for analyses. This study was deemed exempt by the Johns Hopkins Medicine IRB.

### Race/ethnicity and income

Main independent variables based on 2018 US Census data include county-level race/ethnicity (percentage White (ref.), Black, Hispanic, Asian, Native American/Alaskan Native, and two or more races) and the percentage living below the poverty threshold. As in prior work [14, 22], poverty was selected as a socioeconomic measure rather than median household income because the two were highly correlated (ρ = − 0.71) and poverty captures both income and household size.

### Additional variables

This study measured county characteristics that may be associated with poverty or race/ethnicity and influence coronavirus risk using data from the 2018 Centers for Disease Control and Prevention (CDC) Social Vulnerability Index, the 2020 Robert Wood Johnson Foundation County Health Indicators, the 2018 U.S. Census, 2018 hospital data from the Kaiser Family Foundation, the 2013 National Center for Health Statistics urban-rural classification scheme, and the COVID Tracking Project. Rurality was ranked based on population size and density as large central metropolitan areas (ref.), large fringe metropolitan, medium metropolitan, small metropolitan, micropolitan, and non-core areas. Age was classified as percentage of population ≤ 17, 18–64 (ref.) and ≥ 65 years. Sex was measured as percentage of male (ref.) and female. Since lower testing in counties with more Black residents or more poverty [23] may contribute to differential misclassification, cumulative coronavirus testing rate (tests/population) and positive testing rate (positive/total results) between January, 22 to May 20 were measured. Economic characteristics included percentage unemployed (aged ≥16 unemployed and looking for work), percentage lacking high school diplomas (among adults aged > 25 years), percentage of households with housing cost burden (paying > 50% of income for housing), percentage of single parent households and percent of people with limited English proficiency (i.e. speak English “less than well”). Housing and transit variables include percentage of crowded households (more people than rooms), percentage of households in multi-unit buildings (≥10 units), and percentage of households without a vehicle. Population health characteristics included prevalence rates for diabetes, obesity and smoking, which are linked with coronavirus outcomes [13, 16, 24, 25]. Health care characteristics included the percentage uninsured (among adults < 65), rates of preventable hospitalizations (discharges for ambulatory care sensitive conditions) and primary care physicians per 100,000 Medicare enrollees and rates of hospitals and ICU beds per 100,000 population.

### Statistical analyses

Hypotheses were tested with negative binomial models in Stata 15 [26]. Separate models examined cumulative incidence and cumulative mortality. Analyses were clustered within states to account for state-level differences including school and business closures. To account for differences in population size, the exposure was set as the total population.

Model 1 characterized county-level disparities in coronavirus by percentages of poverty and race/ethnicity adjusting for age, sex, rurality, days since index case and testing rate. To account for the spread and detection of the disease in the population, the cumulative mortality model additionally adjusted for positive test rate. Interaction terms between race/ethnicity and poverty with rurality were tested to account for potential urban vs. rural differences. Models 2, 3 and 4 tested whether economic characteristics, housing/transit characteristics, and population health/health care characteristics, respectively, were associated with outcomes and whether accounting for them attenuated disparities. Model 2 added percentages of unemployment, without a high school diploma, housing cost burden, single parent households and limited English proficiency to Model 1. Model 3 added percentages of households that were crowded, in multi-unit buildings and without a vehicle to Model 1. Model 4 added rates of diabetes, obesity, smoking, uninsured, preventable hospitalizations, and primary care physicians and, for cumulative mortality included rates of hospitals and ICU beds to Model 1. No model included all independent variables simultaneously because of potentially mediating pathways (Supplemental Fig. 1) [9]. Since data was missing for < 1% of observations for all variables no imputation was done.

### Sensitivity analyses

To evaluate influential observations, analyses excluded counties with incidence or mortality counts or rates exceeding the 99th percentile. To evaluate time invariance of the disparities, interaction terms between race/ethnicity and poverty with days since index case were tested. Since cases identified prior to the CDC approved testing guidelines on March 3rd may have different risk factors (i.e. travel, access to testing) than cases after that date, sensitivity analyses excluded those cases.

## Results

The average coronavirus cumulative incidence rate was 750 per 100,000 and 3069 (98%) of counties had a total of 3,719,594 cases. The average cumulative mortality rate was 20 per 100,000 and 2074 (66%) of counties had a total of 138,485 deaths. County residents were generally predominately non-Hispanic White (average of 84%). Residents of other races were, on average, Black (9%), Hispanic (10%), Native American (2%), Asian (2%) and two or more races (2%). The average poverty rate was 15.6%. On average, counties were predominately comprised of adults aged 18–64 (59%), with smaller proportions of children ≤17 years (22%) and adults ≥65 years (18%). Counties with higher six-month coronavirus rates had higher percentages Black, Hispanic and Asian residents and lower percentages of Native American residents and higher rates of individuals in poverty (Table 1). All other county characteristics differed based on six-month cumulative incidence rate except for percentage of children ≤17 years and rates of primary care physicians and ICU beds (Table 1).

**Table 1:** Selected U.S. county characteristics based on six-month cumulative incidence rate (*n* = 3142)

	Six-month Cumulative Incidence Rate			
	Low (*n* = 1048)	Moderate (*n* = 1047)	High (*n* = 1047)	*p* value^a^
Range of six-month cumulative coronavirus incidence per 100,000	0–281	282–761	761–13,674	
Mean six-month cumulative coronavirus incidence per 100,000 (SD)	149 (79)	487 (136)	1616 (1091)	< 0.001
Mean six-month cumulative coronavirus mortality per 100,000 (SD)	2.7 (5.7)	12.3 (16.8)	44.2 (51.1)	< 0.001
Mean days since index case (SD)	93.5 (36.0)	112.1 (18.8)	117.6 (12.7)	< 0.001
Mean percent in poverty (SD)	14.7 (5.7)	14.3 (5.8)	17.8 (7.2)	< 0.001
Mean percent in each racial/ethnic group (SD)				
White	91.2 (12.1)	87.9 (11.2)	74.2 (19.3)	< 0.001
Black	2.2 (4.0)	6.1 (8.6)	9.7 (19.2)	< 0.001
Hispanic	5.7 (8.5)	8.9 (12.7)	14.3 (17.5)	0.0001
Asian	1.1 (3.1)	1.6 (3.0)	2.0 (2.7)	< 0.001
Native American	3.0 (9.1)	2.0 (5.9)	2.0 (7.8)	0.0022
Two or more races	2.0 (1.9)	2.3 (1.5)	2.1 (1.0)	0.0001
Mean percent in each age group (SD)				
≤ 17 years	21.4 (3.6)	22.3 (3.2)	23.3 (3.4)	0.2957
18–64 years (ref.)	58.1 (4.1)	59.4 (3.7)	60.3 (3.5)	< 0.001
≥ 65 years	20.4 (4.7)	18.2 (4.3)	16.4 (3.8)	< 0.001
Mean percent in each sex group (SD)				
Male (ref.)	50.5 (2.2)	49.9 (1.7)	49.9 (2.8)	< 0.001
Female	49.5 (2.2)	50.1 (1.7)	50.1 (2.8)	< 0.001
Mean percent tested in state (SD)	12.6 (4.2)	12.6 (3.6)	13.1 (3.9)	0.0014
Mean percent of test results that are positive (SD)	9.1 (5.5)	9.9 (4.9)	10.3 (4.8)	< 0.001
Rurality (%)				< 0.001
Large central metropolitan areas (ref.)	5 (0.5)	12 (1)	51 (5)	
Large fringe metropolitan	53 (5)	150 (14)	165 (16)	
Medium metropolitan	70 (7)	146 (14)	156 (15)	
Small metropolitan	84 (8)	149 (14)	125 (12)	
Micropolitan	213 (20)	213 (20)	215 (21)	
Non-core areas	623 (60)	377 (36)	335 (32)	
**Economic characteristics**				
Mean percent unemployed (SD)	5.4 (2.9)	5.3 (2.3)	6.6 (3.1)	< 0.001
Mean percent without high school diploma (SD)	11.4 (5.3)	12.4 (6.0)	16.4 (6.6)	< 0.001
Mean percent of households with housing cost burden (SD)	10.2 (3.5)	10.5 (3.2)	12.4 (3.8)	< 0.001
Mean percent of single parent households (SD)	7.3 (2.7)	8.0 (2.2)	9.7 (2.8)	< 0.001
Mean percent with limited English proficiency (SD)	0.8 (1.5)	1.4 (2.4)	2.9 (3.6)	< 0.001
**Housing and transit characteristics**				
Mean percent crowded households (SD)	2.1 (3.0)	2.1 (1.6)	3.0 (2.3)	0.0155
Mean percent of households without vehicle (SD)	6.2 (5.7)	5.7 (3.0)	7.1 (4.2)	< 0.001
Mean percent of households in multi-unit buildings (SD)	3.3 (4.2)	4.7 (4.9)	6.0 (7.3)	< 0.001
**Population health and health care characteristics**				
Mean preventable hospitalization rate^b^ (SD)	4602 (2044)	4694 (1602)	5272 (1785)	< 0.001
Mean percent uninsured (SD)	10.6 (4.7)	10.7 (5.2)	13.1 (5.13)	< 0.001
Mean diabetes prevalence (SD)	11.6 (3.7)	11.9 (3.9)	12.9 (4.4)	< 0.001
Mean obesity prevalence (SD)	32.2 (5.1)	32.7 (5.1)	33.7 (6.1)	< 0.001
Mean smoking prevalence (SD)	17.3 (3.8)	17.2 (3.4)	17.9 (3.6)	< 0.001
Mean rate of primary care providers^b^ (SD)	54.0 (36.6)	55.4 (37.5)	54.1 (31.5)	0.6089
Mean hospital rate^b^ (SD)	7.6 (11.7)	4.5 (7.7)	3.7 (5.7)	< 0.001
Mean ICU beds rate^b^ (SD)	11.9 (90.2)	12.3 (19.1)	15.9 (20.5)	0.1711

^a^Obtained from ANOVA

^b^Rate is number per 100,000 population

**Fig. 1:**
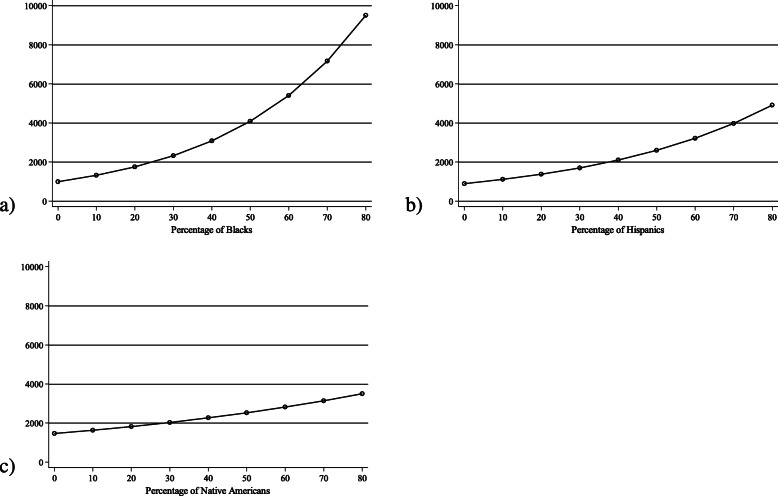
Predicted number of coronavirus cases per U.S. county over six months (*n* = 3142) based on (a) percentage of Blacks or (b) percentage of Hispanics and (c) percentage of Native Americans, obtained from negative binomial model adjusting for age, sex, rurality, days since index case and state testing rate (Model 1, Table 2). Models accounted for clustering within states and used *ln*(population) as the offset

Adjusting for age, sex, rurality, days since index case and testing rate (Model 1, Table 2), each 1 % more Black residents was associated with a 3% (IRR = 1.03, 95% CI: 1.02–1.03) higher risk for each additional coronavirus case and each 1 % more Hispanic residents was associated with a 2% higher (IRR = 1.02, 95% CI: 1.01–1.03) risk. Associations between race/ethnicity and six-month cumulative incidence are depicted in Fig. 1. The poverty rate was not associated with six-month cumulative incidence. Economic and housing characteristics were associated with coronavirus incidence (Models 2 and 3, Table 2). Counties with higher unemployment rates had fewer cases (IRR = 0.96, 95% CI: 0.94–0.98) and counties with more adults lacking high school diplomas had more cases (IRR = 1.03, 95% CI: 1.01–1.05). In models that accounted for economic factors Black and not Hispanic disparities in cumulative incidence persisted (Model 2, Table 2). Counties with higher percentages of multi-unit households had more coronavirus cases (IRR = 1.02, 95% CI: 1.01–1.04). Black and Hispanic disparities in cumulative incidence persisted in models that adjusted for housing/transit characteristics and population health/health care characteristics (Models 3 and 4, Table 2).

**Table 2:** Adjusted associations between U.S. county race/ethnicity, poverty, economic characteristics, housing/transit characteristics, and population health/health care characteristics with six-month cumulative incidence of coronavirus obtained from negative binomial models (*n* = 3142)

	Model 1 (*n* = 3141)	Model 2 (*n* = 3141)	Model 3 (*n* = 3141)	Model 4 (*n* = 2956)
	IRR (95% CI)	IRR (95% CI)	IRR (95% CI)	IRR (95% CI)
Percent in poverty	1.00	0.99	1.01	0.98
	(0.99–1.02)	(0.98–1.01)	(0.99–1.03)	(0.97–1.00)
Race				
Percent White (ref.)				
Percent Black	**1.03**	**1.03**	**1.03**	**1.03**
	**(1.02–1.03)**	**(1.02–1.04)**	**(1.02–1.04)**	**(1.02–1.03)**
Percent Hispanic	**1.02**	1.00	**1.02**	**1.02**
	**(1.01–1.03)**	(0.99–1.02)	**(1.01–1.03)**	**(1.01–1.03)**
Percent Native American	1.01	**1.02**	1.01	1.01
	(1.00–1.02)	**(1.01–1.03)**	(1.00–1.02)	(1.00–1.03)
Percent Asian	1.02	1.00	1.00	1.05
	(0.98–1.05)	(0.96–1.03)	(0.97–1.03)	(1.00–1.09)
Percent two or more races	**0.89**	**0.91**	**0.90**	**0.87**
	**(0.85–0.94)**	**(0.88–0.95)**	**(0.86–0.94)**	**(0.84–0.91)**
Percent unemployed		**0.96**		
		**(0.94–0.98)**		
Percent without a high school diploma		**1.03**		
		**(1.01–1.05)**		
Percent of households with housing cost burden		0.99		
		(0.97–1.02)		
Percent of single parent households		1.01		
		(0.99–1.04)		
Percent with limited English proficiency		1.08		
		(0.99–1.19)		
Percent of crowded households			1.01	
			(0.95–1.08)	
Percent of multi-unit households			**1.02**	
			**(1.01–1.04)**	
Percent of households without vehicle			0.98	
			(0.97–1.00)	
Diabetes prevalence				1.01
				(0.99–1.02)
Preventable hospitalization rate^a^				1.00
				(1.00–1.00)
Percent uninsured				1.02
				(1.00–1.05)
Obesity prevalence				1.00
				(0.99–1.02)
Smoking prevalence				1.02
				(0.99–1.06)
Primary care physician rate^a^				1.00
				(1.00–1.00)

Models accounted for clustering within states and used *ln*(population) as the offset. No data was imputed. In addition to the variables listed above for each model, models adjusted for age, sex, rurality, days since county index case and state testing rate

^a^Rate is number per 100,000 population

In adjusted models, each 1 % more Black (IRR = 1.02, 95% CI: 1.02–1.03) or Native American (IRR = 1.02, 95% CI: 1.01–1.04) residents were both associated with a 2% higher risk of additional coronavirus-related deaths over 6 months (Model 1, Table 3). The poverty rate was not associated with six-month cumulative mortality. Economic and transit characteristics were associated with higher mortality burden, but accounting for them did not attenuate racial disparities in mortality (Models 2 and 3, Table 3). Counties with higher percentages of lacking a high school diploma (IRR = 1.04, 95% CI: 1.01–1.07; Model 2), households with limited English proficiency (IRR = 1.09, 95% CI: 1.04–1.14; Model 2) and households without a vehicle (IRR = 1.04, 95% CI: 1.01–1.07; Model 3) had more coronavirus-related deaths.

**Table 3:** Adjusted associations between U.S. county race/ethnicity, poverty, economic characteristics, housing/transit characteristics, and population health/health care characteristics with six-month cumulative coronavirus-related mortality obtained from negative binomial models (*n* = 3142)

	Model 1 (*n* = 3141)	Model 2 (*n* = 3141)	Model 3 (*n* = 3141)	Model 4 (*n* = 2956)
	IRR (95% CI)	IRR (95% CI)	IRR (95% CI)	IRR (95% CI)
Percent in poverty	1.00	0.97	1.00	0.98
	(0.98–1.02)	(0.95–1.00)	(0.97–1.02)	(0.95–1.01)
Race				
Percent White (ref.)				
Percent Black	**1.02**	**1.02**	**1.02**	**1.03**
	**(1.02–1.03)**	**(1.01–1.03)**	**(1.01–1.03)**	**(1.02–1.03)**
Percent Hispanic	1.00	0.98	1.01	**1.02**
	(1.00–1.01)	(0.98–0.99)	(1.00–1.02)	**(1.01–1.03)**
Percent Native American	**1.02**	**1.03**	**1.03**	**1.04**
	**(1.01–1.04)**	**(1.01–1.04)**	**(1.01–1.04)**	**(1.02–1.05)**
Percent Asian	1.05	1.03	1.04	**1.06**
	(1.00–1.10)	(0.99–1.08)	(1.00–1.09)	**(1.01–1.11)**
Percent two or more races	**0.86**	**0.87**	**0.86**	**0.87**
	**(0.78–0.94)**	**(0.80–0.95)**	**(0.79–0.94)**	**(0.79–0.96)**
Percent unemployed		0.99		
		(0.96–1.02)		
Percent without a high school diploma		**1.04**		
		**(1.01–1.07)**		
Percent of households with housing cost burden		1.00		
		(0.96–1.04)		
Percent of single parent households		1.05		
		(1.00–1.10)		
Percent with limited English proficiency		**1.09**		
		**(1.04–1.14)**		
Percent of crowded households			0.95	
			(0.89–1.01)	
Percent of multi-unit households			1.00	
			(0.98–1.01)	
Percent of households without vehicle			**1.04**	
			**(1.01–1.07)**	
Diabetes prevalence				1.01
				(0.99–1.03)
Preventable hospitalization rate^a^				1.00
				(1.00–1.00)
Percent uninsured				**0.96**
				**(0.93–0.98)**
Obesity prevalence				0.99
				(0.97–1.01)
Smoking prevalence				1.05
				(0.98–1.13)
Primary care physician rate^a^				1.00
				(1.00–1.00)
Hospital rate^a^				0.98
				(0.97–1.00)
ICU bed rate^a^				1.00
				(1.00–1.00)

Models accounted for clustering within states and used *ln*(population) as the offset. No data was imputed. In addition to the variables listed above for each model, models adjusted for age, sex, rurality, days since county index case, state testing rate and percentage of positive test results

^a^Rate is number per 100,000 population

There were no interactions between poverty or race/ethnicity with days since index case. Inferences were unchanged with two additional sensitivity analyses (results not shown), excluding counties with cumulative incidence counts or rates exceeding the 99th percentile and restricting analyses to the 3,296,845 cases identified since CDC guidelines were issued on March 3rd.

## Discussion

During the first 6 months of the U.S. coronavirus epidemic, counties with higher rates of Black or Hispanic residents had higher cumulative incidence and counties with higher rates of Black or Native American residents had higher cumulative coronavirus-related mortality. These results are consistent with those of other studies reviewed earlier showing coronavirus disparities based on Black race and Hispanic ethnicity and build on prior work by identifying disparities based on proportion of Native American residents. Prior studies have documented disparities for multiple other health outcomes [27, 28] for Native American communities and these results show the emergence of coronavirus disparities within the first 6 months of the epidemic. Importantly, this study found no evidence that poor underlying population health accounts for county-level coronavirus disparities. However, results are consistent with Fundamental Cause Theory [9] and Ecosocial Theory [19] in showing that economic, housing and transit characteristics are associated with cumulative incidence and mortality, suggesting they are relevant to disparities in the pandemic.

Structural discrimination may theoretically underlie the results found in this study; discrimination contributes to relatively higher exposure to stressful environments and stressful experiences and relatively less access to health-promoting resources for racial and ethnic minorities than Whites [10, 19]. Structural discrimination can take many forms including racial residential segregation and discriminatory practices that create differential access to educational, economic and health care opportunities based on race or ethnicity [19]. Based on Fundamental Cause Theory [9] and Ecosocial Theory [19], structural discrimination is believed to be a fundamental determinant of health, meaning it is believed to contribute to health through multiple intervening pathways [9, 10, 19]. These pathways likely include both social determinants of health, such as housing and workplace environments that influence exposures, as well as health behaviors and psychosocial factors such as stress exposure and social capital [10, 19]. Importantly, the intervening pathways may differ for Blacks, Hispanics and Native Americans due to differences in historical conditions and current economic, political and social factors [10, 19, 28, 29]. As examples, there is good evidence that racial residential segregation, which has disproportionately affected those who are Black [10], is associated with higher coronavirus incidence in counties with relatively more Black residents [4]. However, Native American groups have a unique history of forced relocations to rural counties lacking economic opportunities and health care access [28] and these conditions may be responsible for the finding in this study that counties with relatively more Native Americans have higher mortality burden despite not having higher incidence burden. Likewise, some Hispanic groups in this country have unique barriers to health care and social services and disproportionately die from preventable causes [29]; this may explain why counties with higher percentage of Hispanic residents were found to have higher mortality burden after accounting for the underlying health of the population. Together with other findings, these results suggest that although coronavirus disparities exist for those who are Black, Hispanic or Native American, the intervening mechanisms may differ in subtle but important ways.

In this study, county economic characteristics including low high school graduation rates and high rates of limited English proficiency were associated with higher coronavirus incidence. These results are consistent with other studies showing higher all-cause mortality related to lower educational attainment [30] and poorer health related to limited English proficiency [31]. Importantly, since educational achievement and English proficiency have both been associated with poorer access to health care [32, 33] these factors may increase coronavirus risk by being barriers to health information and preventive care, in addition to being barriers to economic opportunities. Contrary to our hypothesis, a higher unemployment rate was associated with fewer cases. Unanticipated results may be partly due to working fewer hours during the pandemic rather than an actual protective effect on health, since unemployment [34] has been consistently associated with higher all-cause mortality risk at the individual level. Combined with data elsewhere showing a tripling of the unemployment rate in April 2020 [35], these results suggest that there may be important feedback loops between economic constraints and coronavirus which could accelerate disparities over time for counties lacking economic opportunities. Together, these results suggest that timely intervention is needed to identify economically vulnerable areas and provide targeted support so that communities are not doubly jeopardized by disproportionate coronavirus disease burden and widening economic disparities. Also, these results suggest that additional individual-level data is needed to examine differential occupational risk and workplace exposures based on educational training and employment opportunities.

This study also identified housing and transit characteristics that are associated with coronavirus burden in the population, although they did not fully account for the disparities. This may be because additional intervening pathways link residential racial segregation to health unmeasured in this study, such as discrimination [19], neighborhood conditions and community resources. Results related to lacking a vehicle are consistent with results from a New York City study that found higher coronavirus incidence rates in areas with higher subway ridership [12]. Although household crowding was not associated with coronavirus outcomes in adjusted models in this study, it was associated with coronavirus incidence in another national study [2] and has been associated with higher rates of other respiratory conditions including tuberculosis [36], pneumonia among older adults [37], and respiratory syncytial virus among children [38], suggesting that it may be a relevant target for intervention. These results contribute to the literature by linking multi-unit housing with coronavirus risk, suggesting that either shared spaces within the building or close proximity to neighbors may also be risk factors. Together, results highlight the importance of day-to-day environments related to housing and transit as potential coronavirus risk factors.

Notably, population health and health care access characteristics were not associated with coronavirus incidence or mortality, despite evidence elsewhere that chronic conditions predict coronavirus severity and mortality at the individual level [39, 40]. Together, these results suggest an individual’s risk of dying from coronavirus is higher if they have chronic conditions. However, a county’s coronavirus burden is likely not primarily driven by population health but by the underlying social determinants of health that drive both the chronic conditions and coronavirus outcomes. Therefore, improving health care access may not be sufficient to address pandemic disparities and primary prevention is needed to mitigate disparities. These disparate findings comparing individual-level studies to this population-level study also highlight the need for national reporting of individual-level data. Increasingly, states are releasing racial and ethnic data but these results also show that socioeconomic status is an important risk factor for coronavirus. Such data could not only be useful in tracking individual-level disparities but also be used to design and implement policies and programs that can help vulnerable communities recover after the pandemic. Also, such data could be used with data linkages to test whether chronic conditions and health care access account for individual-level disparities within communities.

This study examined county-level associations; individual-level associations may differ. Although the study selected county characteristics that should be theoretically linked to coronavirus risk and disease severity, additional environmental features of neighborhoods, workplaces, homes and social networks as well as cultural and political factors may also be associated with coronavirus outcomes. This study did not measure hypertension, which predicts coronavirus outcomes [40], but did include related health characteristics [40] including diabetes, obesity and smoking. This study marginalized the effect of state policy changes by clustering within states; additional studies are needed to directly examine state policy changes. This study was strengthened by using all available coronavirus case and mortality data across the U.S. and included all U.S. counties.

These results can help policy makers and public health officials develop strategies to prevent incidence and reduce mortality in vulnerable communities for the current pandemic and future disease outbreaks. As examples, coronavirus testing and contact tracing capacity could be increased in counties with higher rates of individuals who are Black, Hispanic or Native American. Community leaders should be trained in all CDC guidelines, including those specific to multi-generational households and public transit use. Public health information should be readily available in multiple languages to address language barriers for individuals who are not proficient in English. Providing free face-masks, testing and transportation to testing may also be useful to reduce financial barriers to prevention and testing in counties with high poverty rates. Also, addressing economic opportunities and constraints in communities as well as housing and public transit conditions may mitigate disparities. As examples, temporary housing could isolate sick individuals living in crowded housing or multi-unit buildings. Cities could increase the frequency and/or number of vehicles on public transit routes to enable social distancing and establish widespread public transit mask use policies. Economic factors including enforced regulations for paid sick leave, workplace social distancing and personal protective equipment may protect low-wage employees. Importantly, specific additional strategies may be needed to protect individuals working outside of regulated sectors, such as street vendors and sex workers. Economic constraints may be eased for families by increasing the minimum wage to a livable wage, improving access to and benefit amounts for programs including food assistance, cash assistance, unemployment insurance and by increasing access to affordable housing.

## Conclusions

These disparities in coronavirus burden across counties based on race and ethnicity call for greater attention to addressing health equity during and after the pandemic. Importantly, we found that county-level disparities are more related to differential exposure to social determinants of health rather than poor underlying health in the population. Economic conditions, housing conditions and public transit conditions are relevant targets for policies and programs. Addressing these conditions may mitigate the disparities unfolding in vulnerable communities and attenuate disparities for future population health events.

## Supplementary Information

**Additional file 1: Supplemental Figure1**. Directed acyclic graph representing the hypothesized pathways relevant to the association between county poverty rate and county percentage of racial minorities in the U.S.. County economic characteristics measured in this study included percentages of unemployment, without a high school diploma, housing cost burden, single parent households and limited English proficiency. County housing and transit characteristics included percentages of households that were crowded, in multi-unit buildings and without a vehicle. County health and health care characteristics included rates of diabetes, obesity, smoking, uninsured, preventable hospitalizations, and primary care physicians and, for cumulative mortality included rates of hospitals and ICU beds. Since population health characteristics may be caused by economic, housing and transit characteristics and, in turn, influence coronavirus outcomes, models did not adjust for population health and health care characteristics in all models.

## Data Availability

The datasets analyzed during the current study are available from the Robert Wood Johnson Foundation (https://www.countyhealthrankings.org/explore-health-rankings/rankings-data-documentation), the Centers for Disease Control (https://svi.cdc.gov/data-and-tools-download.html), the COVID Tracking Project https://docs.google.com/spreadsheets/u/2/d/e/2PACX-1vRwAqp96T9sYYq2-i7Tj0pvTf6XVHjDSMIKBdZHXiCGGdNC0ypEU9NbngS8mxea55JuCFuua1MUeOj5/pubhtml), Johns Hopkins University & Medicine Coronavirus Resource Center (https://coronavirus.jhu.edu/map.html) and the Kaiser Family Foundation (https://www.kff.org/state-category/providers-service-use/hospitals/).

## Abbreviations

U.S.: United States
CDC: Centers for Disease Control and Prevention
