# Supplementary material for: Race, ethnicity, poverty and the social determinants of the coronavirus divide: U.S. county-level disparities and risk factors
Source: BMC Public Health. 2021 Jun 29;21:1250. doi: 10.1186/s12889-021-11205-w (PMC8240081; doi:10.1186/s12889-021-11205-w)
Supplement: Supplementary file 1 — Additional file 1: Supplemental Figure1. Directed acyclic graph representing the hypothesized pathways relevant to the association between county poverty rate and county percentage of racial minorities in the U.S.. County economic characteristics measured in this study included percentages of unemployment, without a high school diploma, housing cost burden, single parent households and limited English proficiency. County housing and transit characteristics included percentages of households that were crowded, in multi-unit buildings and without a vehicle. County health and health care characteristics included rates of diabetes, obesity, smoking, uninsured, preventable hospitalizations, and primary care physicians and, for cumulative mortality included rates of hospitals and ICU beds. Since population health characteristics may be caused by economic, housing and transit characteristics and, in turn, influence coronavirus outcomes, models did not adjust for population health and health care characteristics in all models. [file 12889_2021_11205_MOESM1_ESM.docx]

Coronavirus incidence/ mortality

County Population Health and Health Care Characteristics

County Economic Characteristics

- County Housing and Transit Characteristics

Percentage of Black, Hispanic, Native American Residents

Percentage in Poverty

Supplemental Figure 1

Directed acyclic graph representing the hypothesized pathways relevant to the association between county poverty rate and county percentage of racial minorities in the U.S.. County economic characteristics measured in this study included percentages of unemployment, without a high school diploma, housing cost burden, single parent households and limited English proficiency. County housing and transit characteristics included percentages of households that were crowded, in multi-unit buildings and without a vehicle. County health and health care characteristics included rates of diabetes, obesity, smoking, uninsured, preventable hospitalizations, and primary care physicians and, for cumulative mortality included rates of hospitals and ICU beds. Since population health characteristics may be caused by economic, housing and transit characteristics and, in turn, influence coronavirus outcomes, models did not adjust for population health and health care characteristics in all models.
